# Contrast enhanced endoscopic ultrasound and detective flow imaging for characterization of gastric neuroendocrine neoplasms

**DOI:** 10.1055/a-2731-6274

**Published:** 2025-11-19

**Authors:** Riccardo Di Pangrazio, Gianluca Esposito, Francesco Panzuto, Marianna Signoretti

**Affiliations:** 1 117698 Department of Digestive Disease, Sant’ Andrea University Hospital, Sapienza University of Rome, Rome, Italy; 2 9311 Department of Medical-Surgical Sciences and Traslational Medicine, Sapienza University of Rome, Rome, Italy

Gastric neuroendocrine neoplasms (GNENs) are rare tumors classified into three types: Type I, the most common typically indolent, is associated with chronic atrophic gastritis (CAG) and hypergastrinemia; type II arises in the setting of Zollinger–Ellison syndrome; type III is sporadic and often aggressive with high metastatic potential 1 .

The European Neuroendocrine Tumor Society guidelines underscore the pivotal role of endoscopic ultrasound (EUS) for GNENs >1 cm (regardless of the type) and for all type III, providing the precise assessment of lesion size, depth of invasion and presence of locoregional lymph nodes. GNENs typically appear as hypoechoic, well-defined, intramural lesions located in the second or third echo layer 2 3 .

Ancillary techniques such as contrast-enhanced EUS (CH-EUS) and detective flow imaging (DFI) enhance B-mode EUS diagnostic capability. CH-EUS allows the real-time evaluation of vascular perfusion after injection of the contrast agent, showing a typical hyperenhancement in the arterial phase followed by early wash-out in low-grade lesions as highlighted for pancreatic NENs 4 . DFI is a novel modality that visualizes low-velocity microvascular flow with higher spatial and temporal resolution than conventional color Doppler 5 .

Case 1 ( Video 1 ): A 72-year-old man with CAG presented with two gastric body polyps. EUS showed homogeneous, hypoechoic submucosal lesions. DFI revealed uniform microvascularization; CH-EUS showed arterial hyperenhancement and early wash-out ( Fig. 1 ). Histology: type I gastric neuroendrocrine tumour (gNET).

CH-EUS and DFI characterization of three gastric neuroendocrine neoplasms, showing vascular patterns.Video 1

**Fig. 1:**
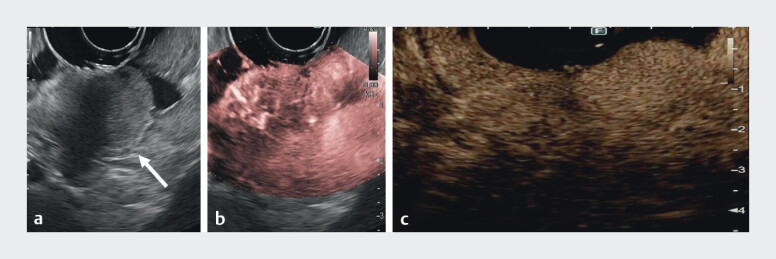
EUS evaluation of a type I gNET. **a** B-mode: hypoechoic homogeneous lesion, limited to the submucosa (arrow). **b** DFI: intralesional microvascularization. **c** CH-EUS: arterial phase hyperenhancement. CH-EUS, contrast-enhanced EUS; DFI, detective flow imaging; EUS, endoscopic ultrasound; gNET, gastric neuroendrocrine tumour.

Case 2 ( Video 1 ): A 75-year-old man without CAG, presented with two polyps in the body. EUS showed inhomogeneous, hypoechoic lesions. DFI showed irregular microvascularization; CH-EUS revealed arterial hyperenhancement and early wash-out ( Fig. 2 ). Histology: type III gNETs.

**Fig. 2:**
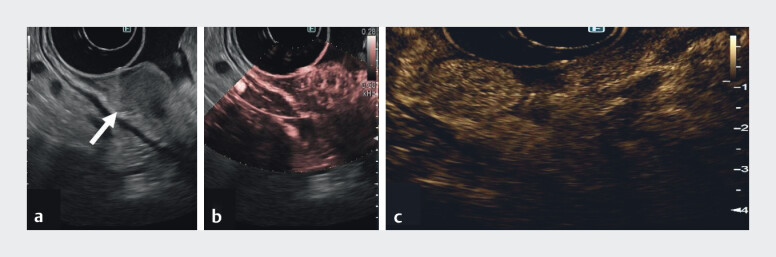
EUS evaluation of a type III gNET. **a** B-mode: hypoechoic inhomogeneous lesion limited to the submucosa (arrow). **b** DFI: intralesional irregular microvascularization. **c** CH-EUS: arterial phase hyperenhancement. CH-EUS, contrast-enhanced EUS; DFI, detective flow imaging; EUS, endoscopic ultrasound; gNET, gastric neuroendrocrine tumour.

Case 3 ( Video 1 ): A 51-year-old man in follow-up for the type I gNET. A new hypoechoic and homogeneous fundic lesion was detected by EUS, with regular microvascularization (DFI) and arterial hyperenhancement (CH-EUS) and early wash-out ( Fig. 3 ). Histology: type I gNET.

**Fig. 3:**
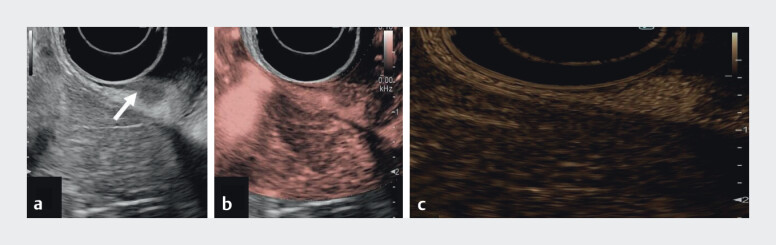
EUS evaluation of a type I gNET. **a** B-mode: hypoechoic homogeneous lesion, limited to the submucosa (arrow). **b** DFI: homogeneous microvascularization. **c** CH-EUS: arterial phase hyperenhancement. CH-EUS, contrast-enhanced EUS; DFI, detective flow imaging; EUS, endoscopic ultrasound; gNET, gastric neuroendrocrine tumour.

These cases illustrate the value of CH-EUS and DFI in the characterization of GNENs.

Endoscopy_UCTN_Code_CCL_1AF_2AD

## Notes

Endoscopy E-Videos https://eref.thieme.de/e-videos
*E-Videos* is an open access online section of the journal *Endoscopy* , reporting on interesting cases and new techniques in gastroenterological endoscopy. All papers include a high-quality video and are published with a Creative Commons CC-BY license. Endoscopy E-Videos qualify for HINARI discounts and waivers and eligibility is automatically checked during the submission process. We grant 100% waivers to articles whose corresponding authors are based in Group A countries and 50% waivers to those who are based in Group B countries as classified by Research4Life (see: https://www.research4life.org/access/eligibility/ ). This section has its own submission website at https://mc.manuscriptcentral.com/e-videos .
